# LIM kinases: cofilin and beyond

**DOI:** 10.18632/oncotarget.16978

**Published:** 2017-04-09

**Authors:** Chloé Prunier, Renaud Prudent, Reuben Kapur, Karin Sadoul, Laurence Lafanechère

**Affiliations:** ^1^ Institute for Advanced Biosciences, INSERM, CNRS UMR, Université Grenoble Alpes, Grenoble, France; ^2^ Department of Molecular Cell Biology, Leiden University Medical Center, Leiden, The Netherlands; ^3^ Cellipse SAS, MINATEC-BHT A441, Grenoble Cedex, France; ^4^ Department of Pediatrics, Herman B Wells Center for Pediatric Research, Indiana University School of Medicine, Indianapolis, IN, USA

**Keywords:** LIM kinases, cytoskeleton, microtubules, inhibitors

## Abstract

LIM kinases are common downstream effectors of several signalization pathways and function as a signaling node that controls cytoskeleton dynamics through the phosphorylation of the cofilin family proteins. These last 10 years, several reports indicate that the functions of LIM kinases are more extended than initially described and, specifically, that LIM kinases also control microtubule dynamics, independently of their regulation of actin microfilament. In this review we analyze the data supporting these conclusions and the possible mechanisms that could be involved in the control of microtubules by LIM kinases. The demonstration that LIM kinases also control microtubule dynamics has pointed to new therapeutic opportunities. Consistently, several new LIM kinase inhibitors have been recently developed. We provide a comprehensive comparison of these inhibitors, of their chemical structure, their specificity, their cellular effects as well as their effects in animal models of various diseases including cancer.

## INTRODUCTION

**Figure 1:**
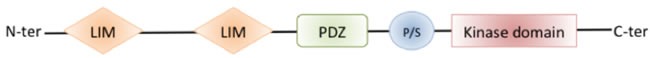
Schematic structure of LIM Kinases

The LIM kinase protein family is composed of two highly related members, LIM kinase 1 (LIMK1) and LIM kinase 2 (LIMK2). Both have the same unique organization of signaling domains, with two amino-terminal LIM domains, adjacent PDZ and proline/serine (P/S)-rich regions, followed by a kinase domain (Figure 1)[1, 2].

Although LIMK1 and LIMK2 are very homologous, particularly within their kinase domain, there is emerging evidence that different upstream pathways can control the activity of each kinase. LIMK1 and LIMK2 may thus contribute to both distinct and overlapping cellular functions [1, 2]. Moreover different patterns of tissue distribution, as well as, for instance distinct localization during cell cycle progression also suggest different biological functions for each kinase [1, 2].

In the present review we will specify LIMK1 or LIMK2 when the data are strictly related to one form of these LIM kinases, whereas we will refer to LIMK when the study does not distinguish between the two isoforms.

It has been shown, using mutants or limited proteolysis, that the LIM domains are able to bind to the C-terminal kinase domain and negatively regulate the kinase activity [3]. The LIM domains also likely contribute to LIMK function by acting as sites of protein-protein interactions, in addition to the PDZ domain.

While LIMK1 and LIMK2 were originally described as serine/threonine kinases, they share sequence similarities with tyrosine kinases [4, 5]. In addition, LIMK1 has been described to present a tyrosine kinase activity [6, 7]. Thus, instead of being strictly serine/threonine kinases, LIMKs appear to have dual-specificity serine/threonine and tyrosine activities.

**Figure 2:**
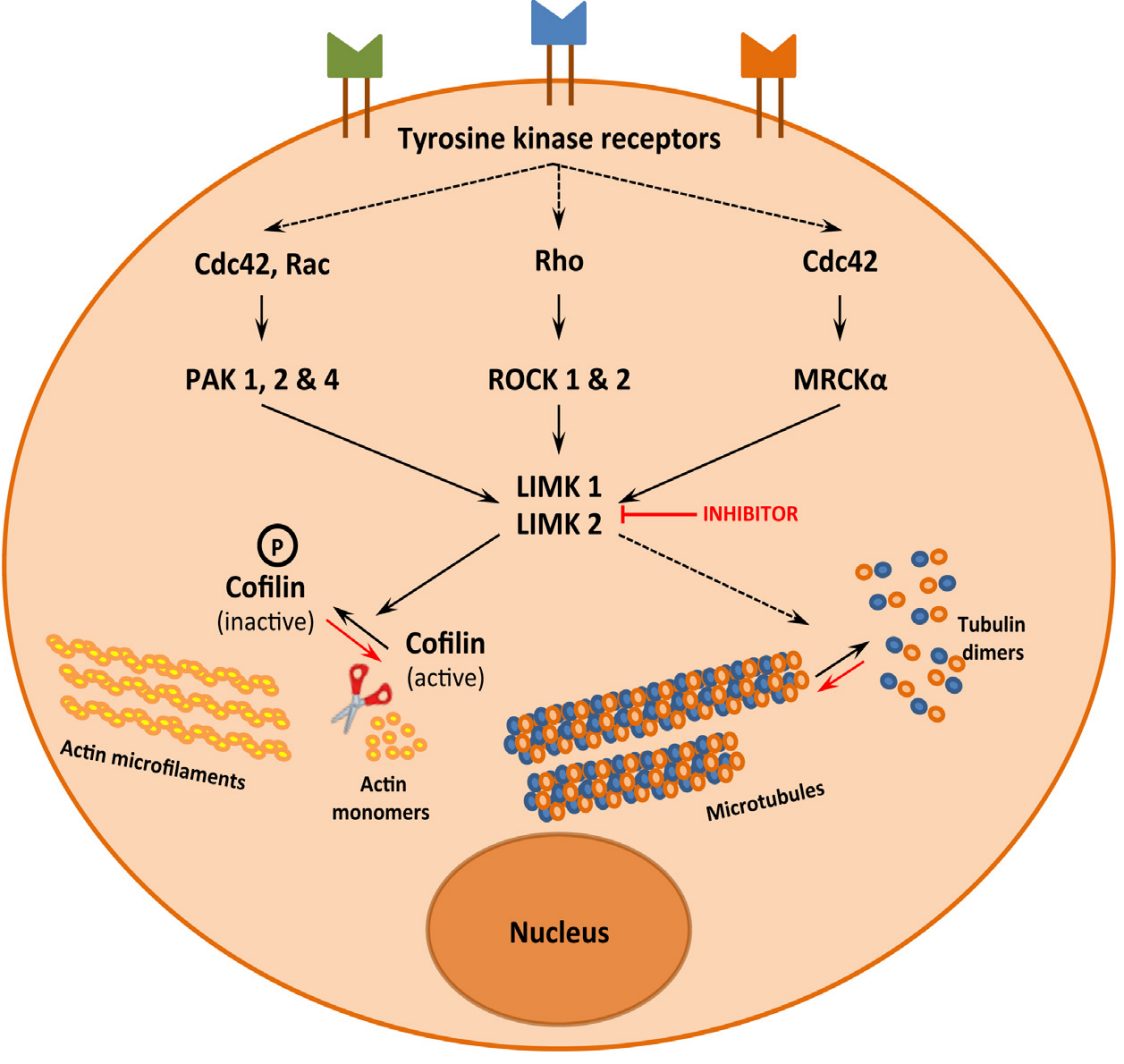
LIMK1 and LIMK2, a signalization hub that controls actin and microtubule dynamics

LIMKs were initially identified as kinases downstream of the Rho pathway (Figure 2). Deregulation of this pathway and of LIMK activity have been implicated in several diseases including cancer and neurological diseases [8–11]. Thus, LIMKs have been described as promising pharmacological targets and a few small molecule inhibitors have been developed [1]. Over the last 5 years, there have been publications indicating that the substrates of LIMK are indeed more diverse than previously thought. Moreover, several new LIMK inhibitors have been reported, which could help to decipher LIMK functions and pave the way for pharmacological applications.

## LIMK: A SIGNALING NODE THAT CONTROLS BOTH ACTIN AND MICROTUBULE DYNAMICS

LIMK was discovered 20 years ago and its initially- and most extensively- described substrates are members of the actin-depolymerizing factor (ADF)/cofilin family of actin-binding proteins. Three forms are expressed in mammals: ADF (also known as destrin); cofilin-1, the major ubiquitous form in non-muscle tissues; and cofilin-2, the major form in differentiated muscle. Although these proteins are different, we will use the general term of cofilin, for the sake of simplicity. Binding of cofilin to actin filaments stabilizes a twisted form of F-actin, thereby weakening lateral subunit interactions and promoting filament severing and depolymerization [12]. However, filament severing by cofilin also results in the generation of free barbed ends, which in turn is crucial for efficient enhancement of actin polymerization [13]. Cofilin is therefore a protein which favors depolymerization or polymerization of actin, depending on the cellular content of actin filaments relative to actin monomers and free barbed ends [14]. Both LIMK1 and LIMK2 phosphorylate and inactivate cofilin at serine 3 allowing an additive fine-tuning of the control of actin dynamics (Figure 2).

Besides LIMK's well-described effect on actin microfilament dynamics through cofilin phosphorylation, several experimental data indicate that LIMK controls microtubule functions, independently of its effect on actin dynamics. Although the mechanism of control of microtubule dynamics by LIMK is still not elucidated, it is now currently admitted that LIMK regulates both components of the cytoskeleton (Figure 2). The following sections aim at summarizing the experimental data that support this conclusion and the possible mechanisms involved in that regulation.

### LIMK regulates microtubule dynamics

Microtubule dynamics can be investigated using several methods. First, modification of the appearance of the microtubule network can be an indicator of the microtubule dynamic status: non dynamic or stabilized microtubules are often bundled or with a sinuous, somehow “curly” appearance [15, 16]. Microtubule dynamics can also be directly followed by measuring the spatial and temporal distribution of microtubule plus-end tracking proteins (+TIPs) that specifically mark growing microtubule plus ends, using immunofluorescence on fixed cells or time-lapse fluorescence microscopy on GFP-+TIP transfected cells. This latter approach allows the analysis of microtubule dynamic instability parameters [17].

The resistance of the microtubule network to cold-[18] or nocodazole-[19] induced depolymerization is also a good indicator of microtubule stability. Indeed, microtubules depolymerize upon cold exposure when not stabilized by associated proteins such as microtubule-associated protein 6 (MAP6, [20]) or by drugs such as paclitaxel [19]. Nocodazole binds free tubulin and prevents its incorporation into microtubules, inducing microtubule depolymerization. Microtubules with slow dynamics have reduced exchanges with the free tubulin pool and are thus less sensitive to nocodazole-induced depolymerization [21].

Finally an increased amount of post-translationally modified tubulin forms is a good marker of microtubule stability. Indeed, it has been shown that old and stable microtubules are enriched in detyrosinated and acetylated tubulin [15]. Thus stabilization of the microtubule network has been revealed using antibodies recognizing detyrosinated [22] or acetylated tubulin [23].

The first work reporting that LIMK regulates microtubule dynamics was published by the group of Voyno-Yasenetskaya, in 2005 [24]. Using immunofluorescence they showed that LIMK1 colocalizes with microtubules in endothelial cells and forms a complex with tubulin through its PDZ domain. Overexpression of LIMK1 induced a depolymerisation of the microtubule network whereas LIMK1 down regulation, using siRNA, induced microtubule stabilization detected by an increase in acetylated microtubules. This required the kinase activity, as LIMK1 kinase-dead mutants were found unable to modify microtubule dynamics. Moreover modulation of LIMK1 expression was sufficient to impact microtubule dynamics, as in these experiments no impact of LIMK2 expression levels was observed [24].

A potential functional link between LIMK2 and microtubule dynamics was however later suggested by M. Kavallaris’ group, when they showed that down regulation of LIMK2 perturbs mitotic spindle formation and causes abnormal cell division [25]. This group also demonstrated that LIMK2 activity is involved in neuroblastoma cell sensitivity to microtubule-destabilizing drugs. Surprisingly, LIMK2 expression is increased in cells selected for resistance to the microtubule destabilizing agents vincristine and colchicine. Thus, down regulation of LIMK2 enhanced cell sensitivity to microtubule-destabilizing drugs, suggesting that LIMK2 mediates sensitivity to these drugs. These results were recently independently confirmed by Mardilovitch and coll. who showed that a pharmacological LIMK inhibitor acts synergistically with agents that inhibit microtubule polymerization, such as vinca alkaloids or colchicine, to inhibit *in vitro* cancer cell proliferation [26].

A comprehensive study about the modification of microtubule dynamics upon LIMK pharmacological inhibition was conducted by Prudent and coll. [19]. This study showed that microtubules treated with the LIMK inhibitor Pyr1 are enriched in detyrosinated tubulin, have reduced dynamic instability and are resistant to nocodazole-induced depolymerization. These stabilizing properties were shared by structurally similar- as well as structurally different- LIMK inhibitors, indicating that they resulted from LIMK inhibition and not from side effects of these pharmacological compounds. Moreover, LIMK1 overexpression was able to counteract the microtubule stabilizing effect of Pyr1 and LIMK down regulation was able to mimic LIMK pharmacological inhibition [19]. It was established that the microtubule stabilizing effect of the LIMK inhibitor was independent of its effect on the actin cytoskeleton because when the microfilaments were completely depolymerized using cytochalasin, Pyr1 was still able to induce the formation of detyrosinated microtubules, indicating that the microtubule network was stabilized.

The signaling axis involving the chemokine CXCL12 and its receptor CXCR4 has been suspected to be involved in the docetaxel chemoresistance of several malignancies, including prostate cancer. Bhardwaj and coll. have shown that stimulation of the receptor CXCR4 by CXCL12 lead to a PAK4-mediated LIMK activation, which induced a destabilization of microtubules and docetaxel resistance. They have also shown that chemical inhibition of LIMK with the compound LIMKi from Bristol-Myers Squibb (BMS) led to a stabilization of the microtubule network, as assessed by an enhancement of detyrosinated tubulin [27].

Using the same LIMK chemical inhibitor, it was independently shown that such an inhibition induced an hyperstabilization of the microtubules of the mitotic spindle, as assessed by their resistance to cold-induced depolymerisation [28].

Recently, Olson's group, when studying the role of LIMK1 in the nuclear translocation of the androgen receptor, showed that pharmacological inhibition of LIMK, using different LIMK inhibitors, induced an increase of acetylated microtubules in prostate cancer cells, indicative of an enhanced microtubule stability [29]. This was also observed in a human lung adenocarcinoma cell line [26].

In a study aiming at understanding the role of the obscurin-RhoGEF in the formation of microtentacles and in the progression of breast cancer, it was observed that a reduced LIMK activity in MCF10A cells treated with sh-obscurins correlated with an increase of tubulin detyrosination, indicative of microtubule stabilization [30].

Finally, a microtubule stabilizing effect following LIMK down regulation has been described *ex vivo* in mouse submandibular salivary glands [31]. Moreover, pharmacological inhibition of LIMK was able to induce a stabilization of microtubules *in vivo* in experimental tumors, as revealed by an increased detyrosination or acetylation [32].

### LIMK regulates mitotic spindle structure and positioning

Besides the above-described effects of LIMK on microtubule dynamics in interphase cells, several groups have reported that LIMK regulates microtubule organization in mitotic spindles.

An early study conducted by Sumi and coll. indirectly suggested a link between LIMK and mitotic microtubules. Using specific antibodies that they raised against LIMK1 and LIMK2, they observed that LIMK1 and LIMK2 underwent a remarkable redistribution in HeLa cells during the cell cycle. LIMK1, which was associated with cell-cell adhesion sites during interphase, concentrated at spindle poles in metaphase cells and then, to the contractile ring during cytokinesis. LIMK2, which was distributed diffusely in the cytoplasm, associated with the mitotic spindle in mitosis. These results indicated that LIMK1 and LIMK2 might have roles in the organization of the mitotic apparatus. Moreover, Sumi and coll. suggested that LIMK2 might have a different role in the control of mitotic spindle organization compared to LIMK1 and could have other targets than cofilin [33].

Later on, a RhoA-ROCK-LIMK2 pathway was found crucial for the regulation of astral microtubule formation as well as spindle orientation [34]. Using wild type and constitutively active (S3A) cofilin mutants, as well as overexpression of slingshot, a phosphatase for cofilin, it was clearly shown that cofilin was not involved in this process. TPPP/p25, a protein that belongs to the tubulin polymerization promoting protein (TPPP) family was suggested to be an alternative substrate of LIMK2 responsible of that process, but this is not the case as it was later shown that TPPP/p25 is rather a substrate of ROCK than of LIMK [35]. Thus the mechanism through which LIMK2 regulates astral microtubules is still unsolved.

Abnormalities in mitotic spindle structure or in spindle positioning was recurrently reported whether LIMK was down regulated using siRNA [36] or inhibited using pharmacological compounds [19, 26].

### Potential control mechanisms of microtubule dynamics by LIMK

Thus, converging evidences indicate that LIMK is able to increase microtubule dynamics and to control microtubule functions. As the microtubule and actin microfilament networks are highly interconnected, this could be an indirect consequence of the regulation of actin dynamics through LIMK mediated cofilin phosphorylation. This is, however, unlikely as it has been observed that complete actin depolymerisation by cytochalasin does not prevent microtubule stabilization induced by LIMK inhibition [19]. Moreover, it has been shown that manipulation of cofilin phosphorylation has no consequence on astral microtubules [34].

**Figure 3:**
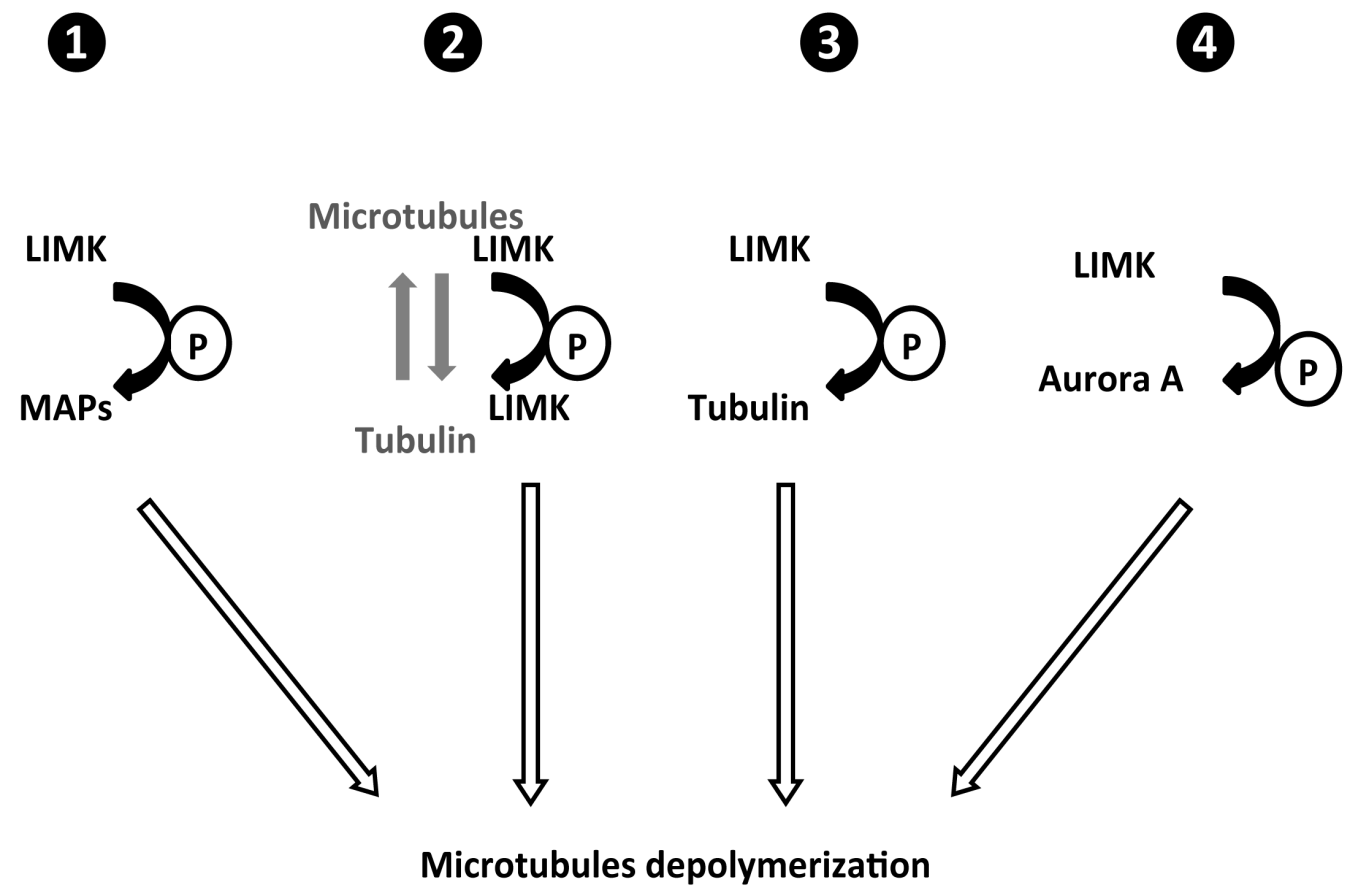
Possible mechanisms for LIMK regulation of microtubule dynamics

The intracellular dynamicity of microtubules is known to be tightly regulated by various microtubule-associated proteins (MAPs) that physically interact with microtubules and promote their stabilization and/or destabilization. The binding of MAPs to microtubules is often regulated by phosphorylations. Although it has been shown that the MAP TPPP/p25 is not a substrate of LIMK [35], as initially thought [37], it could not be excluded that another known or still unidentified MAP could be a substrate of LIMK (Figure 3-1).

Moreover it has been proposed that LIMK1 itself could behave as a MAP: it was shown in prostate cancer cells that LIMK1 physically interacts with tubulin (Figure 3-2). Such an interaction is abolished when the phosphorylation of LIMK1 is increased upon cell stimulation by the cytokine CXCL12. As written by the authors “these data suggest that LIMK1 acts as a MAP in its unphosphorylated state to promote the stability of microtubules” [27].

Likewise, it could not be excluded that LIMK1 directly affects microtubule dynamics by phosphorylating tubulin (Figure 3-3). Such a scenario has been described for CDK1 and β-tubulin, where phosphorylation of tubulin by CDK1 was shown to have consequences in the regulation of microtubule dynamics during mitosis [38].

Finally it has been demonstrated that Aurora-A is a substrate of LIMK [39, 40] and subsequently suggested that Aurora A could be involved in the control of microtubule dynamics by LIMK (Figure 3-4) [41]. Indeed, although Aurora A is a key mitosis regulator, it has been shown that its pharmacological inhibition can impair interphase microtubule dynamics, inducing their stabilisation, raising the issue of the identity of the substrates of Aurora-A that are involved in the regulation of interphase microtubule dynamics [42].

A study regarding megakaryocyte maturation mechanisms has shown that genetic deletion of Pak2 is associated with altered megakaryocyte ultrastructure, including depolymerized microtubule cytoskeleton. Such a phenotype is concomitant with a reduced phosphorylation of LIMK, possibly regulating its activity, and of Aurora-A [41]. The authors conclude that the observed depolymerized microtubule cytoskeleton could result from a reduced Aurora-A activity, which is not in accordance with the suppression of interphase microtubule dynamic instability that would have been expected [42]. Therefore, the interplay between LIMK, Aurora A and microtubule dynamics remains unclear.

Thus, although an increasing amount of data indicates that LIMK is able to regulate microtubule dynamics, independently of their effect on microfilament dynamics, there is still an incomplete understanding of the underlying mechanisms. Approaches aiming at identifying LIMK substrates such as kinase substrate specific labeling [43] would help deciphering such mechanisms.

## OTHER SUBSTRATES OF LIMK: AN UPDATE

Besides cofilin, the transcription factor cAMP-responsive element-binding protein (CREB) was the first other protein that has been reported to be phosphorylated at serine 133 by LIMK1. It was shown that LIMK1 could directly interact with and phosphorylate CREB *in vitro* in immortalized hippocampal progenitor (H19-7) cells and therefore be important for their neuronal differentiation [44]. Recently, these *in vitro* observations, indicating a regulation of CREB by LIMK, have been confirmed *in vivo* using LIMK1 (-/-) mice. The authors showed that such mice have an impaired Long Term Memory (LTM) but a normal short-term memory. They observed that LIMK1 (-/-) mice have a selective defect in late-phase long-term potentiation (L-LTP), a form of synaptic plasticity required for the formation of Long Term Memory (LTM). They showed that LIMK1 regulation of L-LTP is independent of cofilin and that both L-LTP and LTM deficits in LIMK1 (-/-) mice could be rescued by increasing the activity of CREB. Finally they demonstrated that LIMK1 binds to CREB and inhibits its activity through phosphorylation [45].

Besides its role in neuronal functions, CREB has been involved in the control of diverse physiological processes, including the control of cellular metabolism and growth-factor-dependent cell survival. CREB overexpression, which is a marker of worse prognostic in Acute Myeloid Leukemia [46], has been shown to promote abnormal proliferation and survival of myeloid cells [47, 48]. Thus, modulation of CREB activity through LIMK inhibition could represent a therapeutic opportunity.

The nuclear receptor related 1 protein (NURR1) is also known as NR4A2 (nuclear receptor subfamily 4, group A, member 2). The differential activation of NR4A target genes, depending of the cell context, has been described to regulate cell cycle, apoptosis, inflammation, atherogenesis, metabolism, DNA repair and tumorigenesis as well as the midbrain dopamine neuron development, differentiation, and survival [49]. Because of their potential therapeutic interest, both in cancer and in Parkinson disease [49], NURR1 regulators are the subjects of intense research. However, since the original description that LIMK1 binds NURR1 and that LIMK1 overexpression reduced NURR1 transcriptional activity, no new data regarding, for instance, NURR1 phosphorylation by LIMK1 have been reported, to our knowledge.

TPPP/p25 is a MAP that induces tubulin polymerization and microtubule bundling. Microtubule stabilization induced *in vitro* by TPPP/p25 has been shown to be regulated by phosphorylation [50]. It has been claimed that LIMK phosphorylates TPPP/p25 and inhibits microtubule polymerization [34, 37], which could give a molecular explanation of the observed regulation of microtubule dynamics by LIMK. As stated above, this observation turned out to be wrong as it was subsequently clearly demonstrated that TPPP/p25 is in fact phosphorylated by ROCK1 and that neither overexpression of LIMK nor its suppression has an effect on TPPP/p25 phosphorylation. Thus, as ROCKs strongly interact and copurify with LIMK, the previously published findings are likely to reflect TPPP/p25 phosphorylation through contaminant ROCKs [35].

An early study has reported that LIMK1 can autophosphorylate *in vitro* on serine and tyrosine residues [51]. This was confirmed later on, still *in vitro*, by the observation that LIMK1 was able to incorporate ^32^P, while the kinase dead mutant was not [52]. To date, the *in vivo* relevance of these findings, the sites of autophosphorylation and how they may regulate LIMK activity or function are still unknown.

Aurora A kinase is a mitotic kinase that regulates initiation of mitosis through centrosome separation and proper assembly of bipolar spindles. Recently, a mutual phosphorylation of Aurora A and LIMK has been reported, leading to a functional cooperativity of the two kinases. It has been shown that LIMK can activate Aurora A through phosphorylation [39, 53]. In turn, Aurora A directly phosphorylates LIMK primarily at serine 307 (LIMK1 only), serine 283 (LIMK2 only), threonine 494 (LIMK2 only) and threonine 505 (LIMK2 only) [40, 53]. It is speculated that LIMK1 phosphorylation on serine 307 would induce a conformational change that makes LIMK1's threonine 508 accessible for a second site phosphorylation [39]. pLIMK1^T508^ colocalizes with Aurora-A and γ-tubulin [54] to the centrosomes during mitosis, suggesting that recruitment of LIMK1 to the centrosomes is necessary for proper spindle formation through modulation of actin filaments. This study demonstrated that functions of both LIMK1 and Aurora-A are important for the integrity and bipolarity of mitotic spindles [39].

Cytoplasmic Polyadenylation Element Binding (CPEB) proteins, a family of RNA binding proteins, are known to regulate synaptic activity and stabilization of memory. The Drosophila CPEB, called Orb2, and its amyloid-like oligomers are required for long-term memory [55]. Orb2 has two isoforms: Orb2A, which is present only in low abundance, and Orb2B, which is an abundant form. Moreover, Orb2A has a very short half-life and the Orb2 interacting protein Transducer of Erb2 (Tob), a known regulator of cellular growth, stabilizes Orb2A. It has recently been shown that Tob is a substrate for LIMK1 and that Orb2 proteins become a substrate of LIMK1 when associated with Tob. The precise site of phosphorylation has not been determined, but LIMK1 Orb2A phosphorylation is supposed to induce a conformational change, leading to an increased Orb2A half-life, which can act as a seed to induce Orb2 oligomerization [56]. Whether the same regulation mechanism occurs in mammals is not yet known.

Membrane-anchored Type 1- Matrix Metallo Proteinase (MT1-MMP) is a matrix-degrading protease, which is involved in the dissemination of carcinoma cells. It has recently been shown that MT1-MMP and LIMK1 interact in cells through the cytoplasmic part of MT1-MMP and that LIMK1 phosphorylates *in vitro* MT1-MMP at tyrosine 573. This finding confirms that LIMK is not a strict serine/threonine kinase, as well as previous data indicating that LIMK and MT1-MMP functionally interact [57]. It was further shown, using cells knocked down for LIMK1, LIMK2 or both, as well as using a LIMK specific inhibitors, that LIMK activity is required for MT1-MMP-mediated matrix proteolysis [58].

Moreover, although both knockdown of LIMK1 and LIMK2 inhibit matrix degradation, depletion of LIMK1 specifically affects cortactin association on MT1-MMP-positive endosomes while LIMK2 knockdown specifically affects the invadopodial cortactin pool, suggesting non-redundant roles for LIMK1 and LIMK2 in matrix degradation and protein recruitment to invadopodia in breast tumor cells [58].

## PHARMACOLOGICAL INHIBITORS OF LIMK: UNIQUE TOOLS FOR DECIPHERING LIMK FUNCTIONS AND POTENTIAL THERAPEUTICS FOR THE TREATMENT OF DIVERSE PATHOLOGIES

The reaction of phosphorylation catalyzed by kinases such as LIMK allows the cell to rapidly switch off or on the function of the substrates. Regarding LIMK, the relevant substrates regulate highly dynamic polymers such as actin microfilaments and microtubules. Microtubule and actin filament rearrangements typically occur over seconds, a time scale unreachable by the mean of genetic approaches. A clear image of the functions of LIMK needs thus appropriate tools. Small molecules such as pharmacological inhibitors are valuable probes to study dynamic biological processes. Generally acting within minutes or even seconds, they can provide a high degree of temporal control over protein function. As they are also often reversible, they allow both rapid inhibition and re-activation, when withdrawn. Moreover, small molecules permit dose-dependent control of biological functions. If cell-permeable, coupled with approaches such as videomicroscopy, small molecules can give important insights regarding LIMK functions in a cellular context. Indeed, small-molecule inhibitors have proven to be essential in dissecting mechanisms such as mitosis or cytokinesis [59].

Also, small-molecule inhibitors could offer some complementary advantages to LIMK knock down using RNA interference (RNAi). The comparison of their cellular effect to those of RNAi should help to decipher which LIMK functions rely on the kinase domain and which depend on other domains, such as the LIM and PDZ domains.

Moreover, RNAi approaches are not or poorly suitable for some specific cells such as platelets or neurons. Yet LIMK activity plays a central role in platelet maturation [41, 60] and activation [61] as well as in neuronal growth cone motility [4, 6, 62] and in synaptic plasticity [9, 44, 63–65].

Finally, LIMK inhibitors could be potential therapeutic agents as a deregulation of LIMK activity has been reported in several diseases including cancers and cancer cell migration/invasion [8, 29, 66–68], Alzheimer's disease [69], schizophrenia [70], neurofibromatosis type 2 [53], psoriatic epidermal lesions [71], primary pulmonary hypertension [72], allergic diseases [73], ocular hyper-tension and glaucoma [74, 75], erectile dysfunction [76], HIV and other viral infections [77, 78].

Because of the therapeutic potential of LIMK1/ 2 inhibitors, and because of their interest as tools to investigate LIMK functions, the number of reports of new LIMK inhibitors is steadily increasing (Tables 1A, 1B, 1C, 1D).

**Table 1A:** Structure and characteristics of the different published LIMK inhibitors

Structure	IC50 *in vitro*	Selectivity	Activity on cells				Activity on animal models
			Toxicity	Motility and invasion	Actin cytoskeleton	Microtubule cytoskeleton	
***1-*** *N*-{5-[2-(2,6-Dichloro-phenyl)-5-difluoromethyl-2H-pyrazol-3-yl]-thiazol-2-yl}-isobutyramide (aminothiazole scaffold) Named either LIMKi or BMS5 or compound 3 [79] 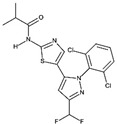	7nM	Profiled against 287 kinases. Inhibits > 76 % ADCK3, ALK4, AMPKA1&2, BRSK1&2,DCAMKL2, FGFR1, PAK3, PCTAIRE1.	No toxicity on A549 cells[79] but has an effect on the proliferation of 4T1.2, MDA MB 213 cells [89] and of androgen dependent prostate cancer cell line [29].	Dose-dependent inhibition of matrigel invasion [90] no effect on MDA MB 231 cell motility [90] but an effect on 4T1.2-pBABE cells [89] as well as prostate cancer cell lines [29].	Inhibits cofilin phosphorylation in A549 cells and in MDA-MB-231 and prostate cancer cell lines[29,79,90]	No detectable change of microtubule network in A549 cells [79] Enhances detyrosinated tubulin in MCF7 cells [19,27] and acetylated tubulin in prostate cancer cell lines [29]	Mouse model: No effect on primary tumor growth nor on metastasis spreading
**2-** Pyrrolopyrimidine derived compounds [75] 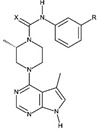	LIMK2: 0.9-3.2 nM LIMK1: 0.5-6.6 nM	Not profiled	Not assayed	Not assayed	50-63 % of inhibition of cofilin phosphorylation in pig trabecular meshwork cells at 10 nM	Not assayed	Decreases intra ocular hypertension in a mouse model, when applied topically
**3-** LX7101 [74] 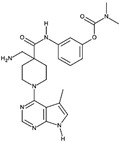	LIMK2 1-7.3 nM LIMK1 32-134 nM	One compound (#28) tested against a panel of 403 kinases showed a moderate selectivity (Kd> 1µM for 34 kinases)	Not assayed	Not assayed	Not assayed	Not assayed	Decreases intra ocular hypertension in a mouse model, when applied topically.

**Table 1B:** Structure and characteristics of the different published LIMK inhibitors

Structure	IC50 *in vitro*	Selectivity	Activity on cells				Activity on animal models
			Toxicity	Motility and invasion	Actin cytoskeleton	Microtubule cytoskeleton	
**4-** Pyridocarbazolone, Pyr1 [19] 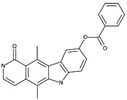	LIMK1 50 nM LIMK2 75 nM	Inhibits LIMK1 efficiently, out of 111 kinases tested	Toxic for different cell lines, including cell lines that overexpress efflux pumps and taxane resistant cell lines [19,32]	Blocks cell motility and cell invasion in vitro but not in tumors [19,32] Inhibits the MT1-MMP-dependent proteolytic matrix degradation [58]	Complete inhibition of cofilin phosphorylation after incubation of cells with Pyr1 25 µM [19] Blockade of actin dynamics (FRAP analysis) [32]	Stabilizes microtubules	Breast tumor growth arrest and metastatic load reduced when injected daily at 10mg/Kg in a mouse model
**5-** T56-LIMKi, oxazole scaffold [83,84] 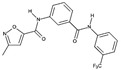	No information	Inhibits more LIMK2 than LIMK1 in cells	Inhibits the proliferation of NF1-/- MEFs [83] and of a pancreatic (Panc-1) cell line as well as glioma and schwannoma cell lines (U87, and ST88-14), but moderate effect on lung (A54) cells[84]	Inhibits wound closure (WT and NF1-/-) [83] Inhibits anchorage-independent growth of NF1-/- MEFs[83]	40% inhibition of cofilin phosphorylation when applied at 50 µM [83,84] Reduction of 26 % of the number of cells exhibiting stress fibers [83].	Not assayed	Dose- and time-dependent decrease pancreatic tumor in a mouse model [84]
**6-** Damnacanthal [7] 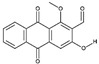	LIMK1: 800 nM LIMK2:1530 nM	Inhibits also Lck, but no Src, ROCK, PAK3, PKCα, CAMK1α [7] Potent inhibitor of p56Ick tyrosine kinase Multikinase inhibitor [82]	Not assayed	Inhibits chemotactic migration of Jurkat cells and Lck-deficient JCaM1.6 cells Inhibits migration and invasion of MDA-MB-231 cells	Inhibits LIMK1-induced deceleration of actin retrograde flow 80% inhibition of cofilin phosphorylation at 10 µM	Not assayed	Inhibits hapten-stimulated migration of epidermal Langerhans cells in mouse ears, when applied topically

**Table 1C:** Structure and characteristics of the different published LIMK inhibitors

Structure	IC50 *in vitro*	Selectivity	Activity on cells				Activity on animal models
			Toxicity	Motility and invasion	Actin cytoskeleton	Microtubule cytoskeleton	
**7-** 4-Aminobenzothieno [3,2-*d*]pyrimidines [87] 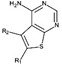	660-1800 nM	Not reported	Not assayed	Not assayed	Not assayed	Not assayed	Not assayed
**8-** aminothiazole pyrimidines [80] 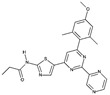	LIMK1 58 nM LIMK2 38 nM	TESK1: 455 nM TESK2: 252 nM At 10 µM, significant binding of only 8 kinases out of a panel of 307 kinases	Inhibition of Thymidine incorportation in HT-1080 cells: IC50 >50µM	Not assayed	Inhibition of cofilin phosphorylation in HT-1080 cells (3-10 µM) Stress fibres depolymerisation in HT-A080 cells (5 µM, 2 days)	Not assayed	Not assayed
**9-** Sulfonamide derivative [81] 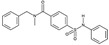	LIMK1: 3,200 nM LIMK2: 39 nM	Only Abl (5,600 nM) and Lck (10,900 nM) are inhibited, out of 24 kinases representative of the 7 kinases families	Not assayed	Not assayed	Not assayed	Not assayed	Not assayed

**Table 1D:** Structure and characteristics of the different published LIMK inhibitors

Structure	IC50 *in vitro*	Selectivity	Activity on cells				Activity on animal models
			Toxicity	Motility and invasion	Actin cytoskeleton	Microtubule cytoskeleton	
**10-** *Bis*-Aryl Urea Derivatives [88] 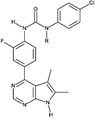	LIMK1: 8-21 nM	Good selectivity against ROCK-I, JNK3 Profiling of the derivatives (18b, 1.0 µM) showed that it inhibits only Limk1, STK16 Aurora-a, Flt3, LRRK2, RET out of 61 kinases.	Not assayed	Invasion of PC-3 cells (Matrigel assay) as well as wound closure are significantly inhibited by a treatment with inhibitors	Inhibit cofilin phosphorylation in A7r5, PC-3 and CEM-SS T cells with an IC50 in the range of hundred nM	Not assayed	Pharmacokinetics studies on rat: reasonable volume of distribution values and reasonable oral bioavailability for some of the compounds Reduction of intraocular pression when topically applied
**11-** CRT0105446 [26,86] 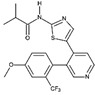	LIMK1: 8 nM LIMK2: 32 nM	Inhibits 51 additional kinases out of 442, at 10 µM	Analysis on 656 cell lines: variable toxicity depending on the cell lines	Blocks invasion of MDAMB231 cells in matrigel	Inhibits cofilin phosphorylation (A 549 cells and MDAMB231 cells)	Induces an increase in acetylated tubulin (A549 cells)	
**12-** CRT0105950 [26,86] 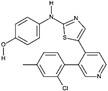	LIMK1:0.3 nM LIMK2: 1 nM	Inhibits 213 additional kinases out of 442, at 10 µM	Analysis on 656 cell lines: variable toxicity depending on the cell lines	Blocks invasion of MDAMB231 cells in matrigel	Inhibits cofilin phosphorylation A 549 cells and MDAMB231 cells)	Induces an increase in acetylated tubulin (A549 cells)	

The most studied inhibitor, LIMKi (also called BMS5 or compound 3) is the first that has been disclosed (compound #1, Table 1A). It was developed by BMS and belongs to a series of inhibitors based on an aminothiazole scaffold, that includes many compounds with off-target effects. For example many of these compounds depolymerize microtubules, independently of their effect on LIMK, which was responsible of their cytotoxicity [79]. Among this series, only LIMKi induces both cofilin dephosphorylation [29, 79] and microtubule stabilization [19, 29] in cells, although it induces a slight depolymerisation of microtubules when assayed *in vitro* on tubulin assembly [79].

Four years later, BMS described a new series of thiazole derivatives, as potent LIMK inhibitors (compound #8, Table 1C) [80]. These cell-permeable compounds were found highly selective, as they inhibit only 8 additional kinases out of a panel of 307 kinases. The activity of these compounds regarding microtubule dynamics has not been reported.

Lexicon pharmaceuticals has also developed a LIMK inhibitor (compound #2, Table 1A, [75]) and a dual LIMK- ROCK inhibitor (compound #3, Table 1A, [74]), that were planned to be used topically for the treatment of ocular hypertension and associated glaucoma. More recently, the same group revealed allosteric LIMK2 inhibitors, based on a sulfonamide scaffold (compound #9, Table 1C). This new class of inhibitors appears to be highly specific for LIMK2. Their cell permeability, as well as their effect on the microtubule cytoskeleton, is however, not reported [81]. It has to be mentioned that apart of these latter compounds, other LIMK inhibitors are proven or predicted Type I inhibitors.

Using a cell-based assay and by screening a diverse chemical library for its ability to modify microtubule dynamics, a pyridocarbazolone, Pyr1, was selected (compound #4, Table 1B). It was further discovered that this compound was a LIMK inhibitor. Although ATP-competitive, Pyr1 inhibits only LIMKs out of 110 kinases tested. When applied on cells, this inhibitor stabilizes microtubules and, through inhibition of cofilin phosphorylation, blocks actin microfilament dynamics [19]. It was further shown that Pyr1 was active on paclitaxel sensitive and resistant tumors, while being well tolerated [19, 32]. Due to its four ring scaffold, this inhibitor is however poorly soluble, limiting its further pharmacological development.

Using a smart fluorescence complementation assay and by screening a small chemical library, a natural product, Damnacanthal (compound #6, Tables 1B) was found to be a cell-permeable LIMK inhibitor [7]. Yet, this compound irreversibly binds LIMKs. Moreover, subsequent studies demonstrated that this compound was a multi kinase inhibitor, with poor selectivity [82].

A LIMK2 inhibitor, T56-LIMKi (compound #5, Table 1B), was discovered through a computer-based procedure by Tel-Aviv University [83]. Although this cell permeable compound showed a high selectivity for LIMK2 versus LIMK1, its effect on other kinases has not been investigated. T56-LIMKi was further shown to inhibit the growth of pancreatic tumor cells in a mouse xenograft model [84].

Using a luciferase based assay of ATP consumption [85], several compounds were selected out of a 60, 000 compound library by high throughput screening and optimized by researchers from the Beatson Institute in collaboration with Cancer Research Technology Discovery Laboratories, UK [26, 86]. These compounds (compound #11 and compound #12, Table 1D) appear to be cell permeable and to have an effect on actin and microtubule dynamics. Although active on LIMK in the nanomolar range, the selectivity of these compounds was variable, when assayed in the micromolar range, depending on the structure of the compounds.

5, 6-substituted 4-aminothieno [2, 3-d]pyrimidines, selected from the same high throughput screening campaign, were also optimized by a group of scientists from Australia. They were found to inhibit LIMK1 in the low micromolar range (compound #7, Table 1D, [87]). The selectivity of these compounds is however unknown, as well as their activity in a cellular context.

Recently, cell-permeable bis-aryl urea derivatives were discovered and optimized by chemists from the Scripps Research Institute in the US. These compounds inhibit potently LIMK. They appear to be selective for LIMK, but they were only assayed for they inhibitory effect on ROCK and JNK kinases (compound #10, Table 1D, [88]).

Thus, the past 5 years have led to the discovery of several structurally different LIMK inhibitors with different characteristics, which may or may not be suitable, depending on their intended use. For instance, inhibitors designed to be used in cells for the understanding of the function of LIMK have to be both highly specific and cell permeable. The need of specificity is less critical for compounds intended to serve as pharmacological drugs, as multi-target kinases have proven to be more efficient in the treatment of cancer. In that latter case, the knowledge of the compound toxicity when administered to animals is crucial.

## CONCLUSIONS

Located downstream of several signaling pathways LIMK occupies a strategic position. It integrates the upstream signals by regulating the dynamics of actin and, most probably, that of microtubules. Convergent data indicate that, indeed LIMK regulates microtubule dynamics. A molecular dissection of the mechanisms involved in that regulation is however essential to ascertain that control. LIMK is also able to regulate the degradation of the matrix by phosphorylating MT1-MMP and to control the activity of Aurora kinase A.

Among the problems still unsolved are the knowledge of the respective role of each of the LIMKs and the understanding of how the activity of each kinase is differentially regulated.

The development of optogenetics as well as the controlled genetic ablation of each kinase should help to solve these problems.

## References

[1] Manetti F (2012). LIM kinases are attractive targets with many macromolecular partners and only a few small molecule regulators. Med Res Rev.

[2] Scott RW, Olson MF (2007). LIM kinases: function, regulation and association with human disease. J Mol Med (Berl).

[3] Nagata K, Ohashi K, Yang N, Mizuno K (1999). The N-terminal LIM domain negatively regulates the kinase activity of LIM-kinase 1. Biochem J.

[4] Okano I, Hiraoka J, Otera H, Nunoue K, Ohashi K, Iwashita S, Hirai M, Mizuno K (1995). Identification and characterization of a novel family of serine/threonine kinases containing two N-terminal LIM motifs. J Biol Chem.

[5] Manning G, Whyte DB, Martinez R, Hunter T, Sudarsanam S (2002). The protein kinase complement of the human genome. Science.

[6] Endo M, Ohashi K, Sasaki Y, Goshima Y, Niwa R, Uemura T, Mizuno K (2003). Control of growth cone motility and morphology by LIM kinase and Slingshot via phosphorylation and dephosphorylation of cofilin. J Neurosci.

[7] Ohashi K, Sampei K, Nakagawa M, Uchiumi N, Amanuma T, Aiba S, Oikawa M, Mizuno K (2014). Damnacanthal, an effective inhibitor of LIM-kinase, inhibits cell migration and invasion. Mol Biol Cell.

[8] Manetti F (2012). Recent findings confirm LIM domain kinases as emerging target candidates for cancer therapy. Curr Cancer Drug Targets.

[9] Cuberos H, Vallée B, Vourc'h P, Tastet J, Andres CR, Bénédetti H (2015). Roles of LIM kinases in central nervous system function and dysfunction. FEBS Lett.

[10] Mali RS, Ramdas B, Ma P, Shi J, Munugalavadla V, Sims E, Wei L, Vemula S, Nabinger SC, Goodwin CB, Chan RJ, Traina F, Visconte V (2011). Rho kinase regulates the survival and transformation of cells bearing oncogenic forms of KIT, FLT3, and BCR-ABL. Cancer Cell.

[11] Prudnikova TY, Rawat SJ, Chernoff J (2015). Molecular pathways: targeting the kinase effectors of RHO-family GTPases. Clin Cancer Res.

[12] Galkin VE, Orlova A, Kudryashov DS, Solodukhin A, Reisler E, Schröder GF, Egelman EH (2011). Remodeling of actin filaments by ADF/cofilin proteins. Proc Natl Acad Sci USA.

[13] Ghosh M, Song X, Mouneimne G, Sidani M, Lawrence DS, Condeelis JS (2004). Cofilin promotes actin polymerization and defines the direction of cell motility. Science.

[14] Bamburg JR (1999). Proteins of the ADF/cofilin family: essential regulators of actin dynamics. Annu Rev Cell Dev Biol.

[15] Gundersen GG, Kalnoski MH, Bulinski JC (1984). Distinct populations of microtubules: tyrosinated and nontyrosinated alpha tubulin are distributed differently in vivo. Cell.

[16] Paturle-Lafanechère L, Manier M, Trigault N, Pirollet F, Mazarguil H, Job D (1994). Accumulation of delta 2-tubulin, a major tubulin variant that cannot be tyrosinated, in neuronal tissues and in stable microtubule assemblies. J Cell Sci.

[17] Honore S, Braguer D (2011). Investigating microtubule dynamic instability using microtubule-targeting agents. Methods Mol Biol.

[18] Lieuvin A, Labbé JC, Dorée M, Job D (1994). Intrinsic microtubule stability in interphase cells. J Cell Biol.

[19] Prudent R, Vassal-Stermann E, Nguyen CH, Pillet C, Martinez A, Prunier C, Barette C, Soleilhac E, Filhol O, Beghin A, Valdameri G, Honoré S, Aci-Sèche S (2012). Pharmacological inhibition of LIM kinase stabilizes microtubules and inhibits neoplastic growth. Cancer Res.

[20] Delphin C, Bouvier D, Seggio M, Couriol E, Saoudi Y, Denarier E, Bosc C, Valiron O, Bisbal M, Arnal I, Andrieux A (2012). MAP6-F is a temperature sensor that directly binds to and protects microtubules from cold-induced depolymerization. J Biol Chem.

[21] Florian S, Mitchison TJ (2016). Anti-Microtubule Drugs.

[22] Vassal E, Barette C, Fonrose X, Dupont R, Sans-Soleilhac E, Lafanechère L (2006). Miniaturization and validation of a sensitive multiparametric cell-based assay for the concomitant detection of microtubule-destabilizing and microtubule-stabilizing agents. J Biomol Screen.

[23] Sadoul K, Khochbin S (2016). The growing landscape of tubulin acetylation: lysine 40 and many more. Biochem J.

[24] Gorovoy M, Niu J, Bernard O, Profirovic J, Minshall R, Neamu R, Voyno-Yasenetskaya T (2005). LIM kinase 1 coordinates microtubule stability and actin polymerization in human endothelial cells. J Biol Chem.

[25] Po’uha ST, Shum MS, Goebel A, Bernard O, Kavallaris M (2010). LIM-kinase 2, a regulator of actin dynamics, is involved in mitotic spindle integrity and sensitivity to microtubule-destabilizing drugs. Oncogene.

[26] Mardilovich K, Baugh M, Crighton D, Kowalczyk D, Gabrielsen M, Munro J, Croft DR, Lourenco F, James D, Kalna G, McGarry L, Rath O, Shanks E (2015). LIM kinase inhibitors disrupt mitotic microtubule organization and impair tumor cell proliferation. Oncotarget.

[27] Bhardwaj A, Srivastava SK, Singh S, Arora S, Tyagi N, Andrews J, McClellan S, Carter JE, Singh AP (2014). CXCL12/CXCR4 signaling counteracts docetaxel-induced microtubule stabilization via p21-activated kinase 4-dependent activation of LIM domain kinase 1. Oncotarget.

[28] Oku Y, Tareyanagi C, Takaya S, Osaka S, Ujiie H, Yoshida K, Nishiya N, Uehara Y (2014). Multimodal effects of small molecule ROCK and LIMK inhibitors on mitosis, and their implication as anti-leukemia agents. PLoS One.

[29] Mardilovich K, Gabrielsen M, McGarry L, Orange C, Patel R, Shanks E, Edwards J, Olson MF (2015). Elevated LIM kinase 1 in nonmetastatic prostate cancer reflects its role in facilitating androgen receptor nuclear translocation. Mol Cancer Ther.

[30] Perry NA, Vitolo MI, Martin SS, Kontrogianni-Konstantopoulos A (2014). Loss of the obscurin-RhoGEF downregulates RhoA signaling and increases microtentacle formation and attachment of breast epithelial cells. Oncotarget.

[31] Ray S, Fanti JA, Macedo DP, Larsen M (2014). LIM kinase regulation of cytoskeletal dynamics is required for salivary gland branching morphogenesis. Mol Biol Cell.

[32] Prunier C, Josserand V, Vollaire J, Beerling E, Petropoulos C, Destaing O, Montemagno C, Hurbin A, Prudent R, de Koning L, Kapur R, Cohen PA, Albiges-Rizo C (2016). LIM Kinase Inhibitor Pyr1 Reduces the Growth and Metastatic Load of Breast Cancers. Cancer Res.

[33] Sumi T, Hashigasako A, Matsumoto K, Nakamura T (2006). Different activity regulation and subcellular localization of LIMK1 and LIMK2 during cell cycle transition. Exp Cell Res.

[34] Heng YW, Lim HH, Mina T, Utomo P, Zhong S, Lim CT, Koh CG (2012). TPPP acts downstream of RhoA-ROCK-LIMK2 to regulate astral microtubule organization and spindle orientation. J Cell Sci.

[35] Schofield AV, Steel R, Bernard O (2012). Rho-associated coiled-coil kinase (ROCK) protein controls microtubule dynamics in a novel signaling pathway that regulates cell migration. J Biol Chem.

[36] Kaji N, Muramoto A, Mizuno K (2008). LIM kinase-mediated cofilin phosphorylation during mitosis is required for precise spindle positioning. J Biol Chem.

[37] Acevedo K, Li R, Soo P, Suryadinata R, Sarcevic B, Valova VA, Graham ME, Robinson PJ, Bernard O (2007). The phosphorylation of p25/TPPP by LIM kinase 1 inhibits its ability to assemble microtubules. Exp Cell Res.

[38] Fourest-Lieuvin A, Peris L, Gache V, Garcia-Saez I, Juillan-Binard C, Lantez V, Job D (2006). Microtubule regulation in mitosis: tubulin phosphorylation by the cyclin-dependent kinase Cdk1. Mol Biol Cell.

[39] Ritchey L, Ottman R, Roumanos M, Chakrabarti R (2012). A functional cooperativity between Aurora A kinase and LIM kinase1: implication in the mitotic process. Cell Cycle.

[40] Johnson EO, Chang KH, Ghosh S, Venkatesh C, Giger K, Low PS, Shah K (2012). LIMK2 is a crucial regulator and effector of Aurora-A-kinase-mediated malignancy. J Cell Sci.

[41] Kosoff RE, Aslan JE, Kostyak JC, Dulaimi E, Chow HY, Prudnikova TY, Radu M, Kunapuli SP, McCarty OJ, Chernoff J (2015). Pak2 restrains endomitosis during megakaryopoiesis and alters cytoskeleton organization. Blood.

[42] Lorenzo C, Liao Q, Hardwicke MA, Ducommun B (2009). Pharmacological inhibition of aurora-A but not aurora-B impairs interphase microtubule dynamics. Cell Cycle.

[43] Allen JJ, Li M, Brinkworth CS, Paulson JL, Wang D, Hübner A, Chou WH, Davis RJ, Burlingame AL, Messing RO, Katayama CD, Hedrick SM, Shokat KM (2007). A semisynthetic epitope for kinase substrates. Nat Methods.

[44] Yang EJ, Yoon JH, Min DS, Chung KC (2004). LIM kinase 1 activates cAMP-responsive element-binding protein during the neuronal differentiation of immortalized hippocampal progenitor cells. J Biol Chem.

[45] Todorovski Z, Asrar S, Liu J, Saw NM, Joshi K, Cortez MA, Snead OC, Xie W, Jia Z (2015). LIMK1 regulates long-term memory and synaptic plasticity via the transcriptional factor CREB. Mol Cell Biol.

[46] Cheng JC, Esparza S, Sandoval S, Shankar D, Fu C, Sakamoto KM (2007). Potential role of CREB as a prognostic marker in acute myeloid leukemia. Future Oncol.

[47] Shankar DB, Cheng JC, Kinjo K, Federman N, Moore TB, Gill A, Rao NP, Landaw EM, Sakamoto KM (2005). The role of CREB as a proto-oncogene in hematopoiesis and in acute myeloid leukemia. Cancer Cell.

[48] Pigazzi M, Ricotti E, Germano G, Faggian D, Aricò M, Basso G (2007). cAMP response element binding protein (CREB) overexpression CREB has been described as critical for leukemia progression. Haematologica.

[49] Decressac M, Volakakis N, Björklund A, Perlmann T (2013). NURR1 in Parkinson disease—from pathogenesis to therapeutic potential. Nat Rev Neurol.

[50] Hlavanda E, Klement E, Kókai E, Kovács J, Vincze O, Tökési N, Orosz F, Medzihradszky KF, Ovádi J, Dombrádi V (2007). Phosphorylation blocks the activity of tubulin polymerization-promoting protein (TPPP): identification of sites targeted by different kinases. J Biol Chem.

[51] Pröschel C, Blouin MJ, Gutowski NJ, Ludwig R, Noble M (1995). Limk1 is predominantly expressed in neural tissues and phosphorylates serine, threonine and tyrosine residues in vitro. Oncogene.

[52] Kobayashi M, Nishita M, Mishima T, Ohashi K, Mizuno K (2006). MAPKAPK-2-mediated LIM-kinase activation is critical for VEGF-induced actin remodeling and cell migration. EMBO J.

[53] Petrilli A, Copik A, Posadas M, Chang LS, Welling DB, Giovannini M, Fernández-Valle C (2014). LIM domain kinases as potential therapeutic targets for neurofibromatosis type 2. Oncogene.

[54] Chakrabarti R, Jones JL, Oelschlager DK, Tapia T, Tousson A, Grizzle WE (2007). Phosphorylated LIM kinases colocalize with gamma-tubulin in centrosomes during early stages of mitosis. Cell Cycle.

[55] Krüttner S, Traunmüller L, Dag U, Jandrasits K, Stepien B, Iyer N, Fradkin LG, Noordermeer JN, Mensh BD, Keleman K (2015). Synaptic Orb2A Bridges Memory Acquisition and Late Memory Consolidation in Drosophila. Cell Reports.

[56] Si K, Kandel ER (2016). The Role of Functional Prion-Like Proteins in the Persistence of Memory. Cold Spring Harb Perspect Biol.

[57] Tapia T, Ottman R, Chakrabarti R (2011). LIM kinase1 modulates function of membrane type matrix metalloproteinase 1: implication in invasion of prostate cancer cells. Mol Cancer.

[58] Lagoutte E, Villeneuve C, Lafanechère L, Wells CM, Jones GE, Chavrier P, Rossé C (2016). LIMK Regulates Tumor-Cell Invasion and Matrix Degradation Through Tyrosine Phosphorylation of MT1-MMP. Sci Rep.

[59] Peterson JR, Mitchison TJ (2002). Small molecules, big impact: a history of chemical inhibitors and the cytoskeleton. Chem Biol.

[60] Kauskot A, Poirault-Chassac S, Adam F, Muczynski V, Aymé G, Casari C, Bordet JC, Soukaseum C, Rothschild C, Proulle V, Pietrzyk-Nivau A, Berrou E, Christophe OD (2016). LIM kinase/cofilin dysregulation promotes macrothrombocytopenia in severe von Willebrand disease-type 2B. JCI Insight.

[61] Pandey D, Goyal P, Bamburg JR, Siess W (2006). Regulation of LIM-kinase 1 and cofilin in thrombin-stimulated platelets. Blood.

[62] Montani L, Gerrits B, Gehrig P, Kempf A, Dimou L, Wollscheid B, Schwab ME (2009). Neuronal Nogo-A modulates growth cone motility via Rho-GTP/LIMK1/cofilin in the unlesioned adult nervous system. J Biol Chem.

[63] Meng Y, Zhang Y, Tregoubov V, Falls DL, Jia Z (2003). Regulation of spine morphology and synaptic function by LIMK and the actin cytoskeleton. Rev Neurosci.

[64] Liu A, Zhou Z, Dang R, Zhu Y, Qi J, He G, Leung C, Pak D, Jia Z, Xie W (2016). Neuroligin 1 regulates spines and synaptic plasticity via LIMK1/cofilin-mediated actin reorganization. J Cell Biol.

[65] Wang W, Townes-Anderson E (2016). Lim kinase, a bi-functional effector in injury-induced structural plasticity of synapses. Neural Regen Res.

[66] Shibue T, Brooks MW, Weinberg RA (2013). An integrin-linked machinery of cytoskeletal regulation that enables experimental tumor initiation and metastatic colonization. Cancer Cell.

[67] Wang W, Eddy R, Condeelis J (2007). The cofilin pathway in breast cancer invasion and metastasis. Nat Rev Cancer.

[68] Park JB, Agnihotri S, Golbourn B, Bertrand KC, Luck A, Sabha N, Smith CA, Byron S, Zadeh G, Croul S, Berens M, Rutka JT (2014). Transcriptional profiling of GBM invasion genes identifies effective inhibitors of the LIM kinase-Cofilin pathway. Oncotarget.

[69] Heredia L, Helguera P, de Olmos S, Kedikian G, Solá Vigo F, LaFerla F, Staufenbiel M, de Olmos J, Busciglio J, Cáceres A, Lorenzo A (2006). Phosphorylation of actin-depolymerizing factor/cofilin by LIM-kinase mediates amyloid beta-induced degeneration: a potential mechanism of neuronal dystrophy in Alzheimer’s disease. J Neurosci.

[70] Yin DM, Chen YJ, Lu YS, Bean JC, Sathyamurthy A, Shen C, Liu X, Lin TW, Smith CA, Xiong WC, Mei L (2013). Reversal of behavioral deficits and synaptic dysfunction in mice overexpressing neuregulin 1. Neuron.

[71] Honma M, Shibuya T, Fujii M, Iinuma S, Ishida-Yamamoto A (2017). Aberrant LIM-kinase 1 expression in hyperproliferative psoriatic epidermis. J Dermatol.

[72] Foletta VC, Lim MA, Soosairajah J, Kelly AP, Stanley EG, Shannon M, He W, Das S, Massague J, Bernard O (2003). Direct signaling by the BMP type II receptor via the cytoskeletal regulator LIMK1. J Cell Biol.

[73] Kapur R, Shi J, Ghosh J, Munugalavadla V, Sims E, Martin H, Wei L, Mali RS (2016). ROCK1 via LIM kinase regulates growth, maturation and actin based functions in mast cells. Oncotarget.

[74] Harrison BA, Almstead ZY, Burgoon H, Gardyan M, Goodwin NC, Healy J, Liu Y, Mabon R, Marinelli B, Samala L, Zhang Y, Stouch TR, Whitlock NA (2014). Discovery and Development of LX7101, a Dual LIM-Kinase and ROCK Inhibitor for the Treatment of Glaucoma. ACS Med Chem Lett.

[75] Harrison BA, Whitlock NA, Voronkov MV, Almstead ZY, Gu KJ, Mabon R, Gardyan M, Hamman BD, Allen J, Gopinathan S, McKnight B, Crist M, Zhang Y (2009). Novel class of LIM-kinase 2 inhibitors for the treatment of ocular hypertension and associated glaucoma. J Med Chem.

[76] Cui K, Luan Y, Wang T, Zhuan L, Rao K, Wang SG, Ye ZQ, Liu JH, Wang DW (2017). Reduced corporal fibrosis to protect erectile function by inhibiting the Rho-kinase/LIM-kinase/cofilin pathway in the aged transgenic rat harboring human tissue kallikrein 1. Asian J Androl.

[77] Manetti F (2012). HIV-1 proteins join the family of LIM kinase partners New roads open up for HIV-1 treatment. Drug Discov Today.

[78] Wen X, Ding L, Wang JJ, Qi M, Hammonds J, Chu H, Chen X, Hunter E, Spearman P (2014). ROCK1 and LIM kinase modulate retrovirus particle release and cell-cell transmission events. J Virol.

[79] Ross-Macdonald P, de Silva H, Guo Q, Xiao H, Hung CY, Penhallow B, Markwalder J, He L, Attar RM, Lin TA, Seitz S, Tilford C, Wardwell-Swanson J, Jackson D (2008). Identification of a nonkinase target mediating cytotoxicity of novel kinase inhibitors. Mol Cancer Ther.

[80] He L, Seitz SP, Trainor GL, Tortolani D, Vaccaro W, Poss M, Tarby CM, Tokarski JS, Penhallow B, Hung CY, Attar R, Lin TA (2012). Modulation of cofilin phosphorylation by inhibition of the Lim family kinases. Bioorg Med Chem Lett.

[81] Goodwin NC, Cianchetta G, Burgoon HA, Healy J, Mabon R, Strobel ED, Allen J, Wang S, Hamman BD, Rawlins DB (2014). Discovery of a Type III Inhibitor of LIM Kinase 2 That Binds in a DFG-Out Conformation. ACS Med Chem Lett.

[82] García-Vilas JA, Pino-Ángeles A, Martínez-Poveda B, Quesada AR, Medina MÁ (2017). The noni anthraquinone damnacanthal is a multi-kinase inhibitor with potent anti-angiogenic effects. Cancer Lett.

[83] Mashiach-Farkash E, Rak R, Elad-Sfadia G, Haklai R, Carmeli S, Kloog Y, Wolfson HJ (2012). Computer-based identification of a novel LIMK1/2 inhibitor that synergizes with salirasib to destabilize the actin cytoskeleton. Oncotarget.

[84] Rak R, Haklai R, Elad-Tzfadia G, Wolfson HJ, Carmeli S, Kloog Y (2014). Novel LIMK2 Inhibitor Blocks Panc-1 Tumor Growth in a mouse xenograft model. Oncoscience.

[85] Mezna M, Wong AC, Ainger M, Scott RW, Hammonds T, Olson MF (2012). Development of a high-throughput screening method for LIM kinase 1 using a luciferase-based assay of ATP consumption. J Biomol Screen.

[86] Charles MD, Brookfield JL, Ekwuru TC, Stockley M, Dunn J, Riddick M, Hammonds T, Trivier E, Greenland G, Wong AC, Cheasty A, Boyd S, Crighton D, Olson MF (2015). Discovery, Development, and SAR of Aminothiazoles as LIMK Inhibitors with Cellular Anti-Invasive Properties. J Med Chem.

[87] Sleebs BE, Nikolakopoulos G, Street IP, Falk H, Baell JB (2011). Identification of 5,6-substituted 4-aminothieno[2,3-d]pyrimidines as LIMK1 inhibitors. Bioorg Med Chem Lett.

[88] Yin Y, Zheng K, Eid N, Howard S, Jeong JH, Yi F, Guo J, Park CM, Bibian M, Wu W, Hernandez P, Park H, Wu Y (2015). Bis-aryl urea derivatives as potent and selective LIM kinase (Limk) inhibitors. J Med Chem.

[89] Li R, Doherty J, Antonipillai J, Chen S, Devlin M, Visser K, Baell J, Street I, Anderson RL, Bernard O (2013). LIM kinase inhibition reduces breast cancer growth and invasiveness but systemic inhibition does not reduce metastasis in mice. Clin Exp Metastasis.

[90] Scott RW, Hooper S, Crighton D, Li A, König I, Munro J, Trivier E, Wickman G, Morin P, Croft DR, Dawson J, Machesky L, Anderson KI (2010). LIM kinases are required for invasive path generation by tumor and tumor-associated stromal cells. J Cell Biol.

